# Influence of Gaming Addiction on Psychosocial Well-Being

**DOI:** 10.7759/cureus.101262

**Published:** 2026-01-10

**Authors:** Ritesh G Menezes, Samata Nepal, Mimosa Bajracharya, Asma A Alshammari, Khwaja Moizuddin, Alok Atreya

**Affiliations:** 1 College of Medicine, Imam Abdulrahman Bin Faisal University, Dammam, SAU; 2 Department of Community Medicine, Lumbini Medical College, Palpa, NPL; 3 Department of General Medicine, Lumbini Medical College, Palpa, NPL; 4 Department of Forensic Medicine, Lumbini Medical College, Palpa, NPL

**Keywords:** compulsive gaming, gaming addiction, gaming disorder, pathological gaming, psychosocial aspects

## Abstract

The gaming journey started decades ago, beginning with simple video games and evolving into flexible mobile games that are accessible everywhere and at any time. This review assesses gaming addiction in terms of risk factors and psychosocial consequences. Gaming disorder is a term used to describe a behavioral condition related to abnormal gaming usage. It is now formally acknowledged as a clinical condition by major international diagnostic classifications. Furthermore, gaming addiction is an escalating global problem that affects various aspects of individuals’ lives. Multiple intrinsic and extrinsic factors influence the likelihood of developing gaming addiction. Intrinsic factors include being young, male, or having a pre-existing psychological condition. Extrinsic factors include peer influence and exposure to social media. Moreover, various consequences of gaming disorder can lead to harmful events, either to oneself or others. Suicide is one of the most serious complications, alongside self-harm, homicide, or violent behavior toward others. In addition, neuropsychological effects can play a major role in life dysfunction, impairing learning capabilities and causing memory loss.

## Introduction and background

In the rapidly changing world, innovation is a gift that creates and improves prospects for what is to come, as well as the perils of unexpected hazards. Gaming is naturally not immune to this visual binary. The same game offering so much pleasure and fun can be associated with excessive screen time, social isolation, and a plethora of mental health problems [1]. Gaming disorder is a type of severe behavioral disorder that can impair various areas of functioning [2]. Due to its severe consequences, gaming disorder is recognized as a clinical condition in the 11th revision of the International Classification of Diseases (ICD-11) and addressed in the Diagnostic and Statistical Manual of Mental Disorders (DSM-5) [2]. Gaming addiction manifests as a behavioral disorder, with effects ranging from social withdrawal to severe forensic consequences like theft, suicide, and homicide, as seen in documented cases [1-3]. The impact of excessive gaming is serious, leading to psychological distress and forensic outcomes like suicides and homicides, necessitating urgent action for gamers and communities [2,3].

## Review

Evolution and appeal of gaming

The rapid evolution of technology has really opened up a worldwide universe of academic, information, and entertainment opportunities [4]. From movies to online games and the latest craze in virtual reality, technology has changed how we view entertainment. As such, entertainment is highly sought after, and people crave constant stimulation through diverse experiences [5]. The roots of video games trace back to the 1950s and 1960s with simple computer-based games and simulations, but it was the introduction of arcade video games in the 1970s, starting with Computer Space (1971) by Nolan Bushnell and Ted Dabney, followed by Atari’s wildly successful Pong (1972)-that sparked a profound cultural shift toward digital consumption [6].

With smartphones being our constant companions in this golden age of media, companies have found massive audiences on mobile platforms [5]. They are designed with a seamless fit into our daily lives, specifically created to leverage phone portability and connectivity for both online and offline play [5]. As technology has improved, the games are designed to utilize special features the smartphone has to offer to create a dynamic and enthralling gaming experience [5]. Pokémon Go is a fitting example. The game relies on the phones’ GPS and Augmented Reality (AR) technology that enables users to navigate real-life locations and encounter virtual Pokémon characters superimposed onto their surroundings [7].

On top of being easy to access, mobile games such as role-playing games (RPGs) like Genshin Impact, Dragon Quest series, and Final Fantasy Franchise provide users with an immersive world where players can interact with each other, cross international boundaries, forge friendships, and learning of new skills [5]. Battle Royale games such as Garena Free Fire and PUBG (Player Unknown’s Battlegrounds) are other most popular thrilling games among teenagers these days, because their competitive nature keeps players engaged in strategic thinking for surviving challenges [5]. Alongside these thrilling games, more calming and meditative games are also available, such as Alto’s Adventure, Zen Koi, Hungry Hearts Diner, and Stardew Valley, while puzzling games like Gorogoa and Gardenscapes, are also captivating yet offer relaxing gameplay [5]. Gaming is now a new channel for a quick and easy dopamine fix with numerous alternatives fitting different age groups, as evidenced by the popularity of games such as Candy Crush Saga [8].

Social and professional influences on gaming

Social media and its great power of influence help popularize mobile games [9-11]. This proliferation of entertainment helped spark interest in a virtual online game, ‘Among Us’ [12], made popular when the COVID-19 pandemic broke out around two years after its release; it became widely publicized, with media attention, because ‘Twitch’ streamers and YouTubers started playing it.

Access to the internet, and consequently to both national and international audiences, has led to a rise in gaming streamers and professional gamers [13]. Their popularity and financial support influence many of the younger generation to play games that are deemed popular [13]. Moreover, people also aspire to become professional players due to leagues that are formed in various e-sports for championships or tournaments, which are opportunities for players to show off their skills, compete, gain fame, as well as earn sponsorships, profit through streaming, and win prizes [13].

Factors contributing to gaming addiction

The gripping form of media, a source of personal entertainment, appeals to adult viewers, stimulating them to try gaming themselves, and thus contributing to their gaming addiction. In the case of young children, it is common to witness parents and/or guardians soothe their crying child by giving them their mobile phone or engaging them for a while when they are busy. We currently reside in a digital age where everything a teenage child needs to know is at the touch of their hands. Peer influence drives teenage gaming addiction as a means of social acceptance, compounded by psychological distress like depression, a known risk factor in internet addiction [3,14]. And this eagerness to belong can drive young people, for instance, into gaming as a means by which they feel connected and accepted [15]. In addition, gaming has an appeal over its large and impressionable young fan base by granting “glory” in the form of money or attention [16], that can pave their way to fame and the other accompanying fringe benefits, which signals a slow descent into addiction as players place themselves in pursuit of one victory after another within game time achievements recognized offline [15-17].

Extreme consequences of gaming addiction

Over the last few years, well-publicized cases have highlighted the extreme consequences of gaming addiction, with gamers experiencing apparent psychotic breaks both personally and professionally. The impact of gaming is undeniably terrifying, ranging from self-harm to violent acts against others, as evidenced by specific incidents linked to games like the Blue Whale Challenge and PUBG. From games influencing the players to commit suicides (Blue Whale Challenge that was developed by a former psychology student, led to over 16 deaths) and revenge murders related to issues brought by gaming, the impact of gaming on our lives is undeniably terrifying [18-20].

Suicide

Gaming addiction has been implicated in numerous suicides worldwide, often triggered by game-related distress or restrictions. During the COVID-19 pandemic, there were 3 PUBG-related suicides in Pakistan within a few days of each other [21]. Such events are not bound to a certain country or ethnicity. In India, a 20-year-old man committed suicide after his parents refused to provide a rechargeable internet pack for his mobile phone to continue playing PUBG [22]. Similarly, separate incidents saw a 24-year-old female polytechnic student, a 14-year-old boy, a 15-year-old boy, and a 17-year-old male reportedly commit suicide after being reprimanded for playing PUBG [22].

Homicide

Beyond self-harm, gaming addiction has also fueled violent acts, including homicides with chilling forensic implications. Tragically, PUBG-related murders have also been reported [22]. In two separate cases, a 12-year-old boy was found dead on a road after leaving home, and a 15-year-old boy fatally stabbed his elder brother [22]. Perhaps the most horrifying incident reported is a case where a 21-year-old youth allegedly decapitated his father and mutilated the body [22]. These revenge murders and violent outbursts demonstrate how gaming addiction can turn into catastrophic interpersonal harm, which poses major challenges for forensic analysis and intervention.

India has documented more than 100 deaths related to gaming since 2019, predominantly suicides tied to PUBG addiction, often after parental intervention [23]. These cases involve forensic examination of contributory factors such as impulse control loss. Some of the noteworthy cases that made headlines are tabulated in Table 1 [24-28].

**Table 1:** Selected documented cases of suicide and homicide directly attributed to gaming addiction in India.

Case Type	Case Details	Outcome
Suicide [24]	A 16-year-old class 10 boy hanged himself after being scolded for PUBG overuse. (Chhattisgarh, October 2024)	Abetment to suicide; parents cleared, but highlighted parental negligence
Suicide [25]	A 15-year-old committed suicide after parental scolding related to gaming addiction. (Andhra Pradesh, September 2019)	No charges; sparked calls for de-addiction centers
Suicide [26]	Three youngsters (aged 17–22) committed suicide after India’s PUBG ban. (Gujarat/Karnataka, September 2020)	Government cited “addiction risks” in the ban justification; no developer liability pursued
Homicide [27]	A 16-year-old boy shot his mother over PUBG access denial. (Uttar Pradesh, June 2022)	Minor tried under the Juvenile Justice Act; plea for a game ban in court.
Homicide [28]	A 13-year-old boy was murdered by his neighbor, a minor, following a PUBG addiction-fueled altercation. (Karnataka, April 2021)	Juvenile proceedings; family sued the game publisher (case dismissed).

Impulsive real-life risks and exploitation

Gaming addiction’s social hooks: voice chats, clans, can forge obsessive virtual relationships, prompting dangerous real-world actions. In 2023, a Pakistani national (27-year-old, female) illegally crossed into India via Nepal with her four children to marry an Indian national (22-year-old, male), her PUBG partner met in 2019; arrested for immigration violations, she faced espionage probes and family custody battles [29]. Similarly, in June 2024, an American woman (22-year-old) flew 12,000 km to Uttar Pradesh in India, to meet her PUBG acquaintance (23-year-old, male); detained en route amid trafficking fears, though cleared after visa verification [30]. Social media reports a case in 2024 where a Vietnamese woman traveled to India to meet her Free-Fire partner [31]. In 2019, a Malaysian man left his family, including his pregnant wife, due to PUBG dependency for uninterrupted play; this highlights a case of gaming addiction leading to relational rupture [32].

Gaming addiction as a clinical disorder

The deleterious effects of internet gaming have been recognized, declaring it a clinical disorder. The World Health Organization (WHO) defines it as a “pattern of gaming behavior characterized by impaired control over gaming, increasing priority given to gaming over other activities to the extent that gaming takes precedence over other interests and daily activities, and continued or escalation of gaming despite the occurrence of negative consequences” [17,33]. This recognition has spurred tools such as Saini-Hodgins Addiction Risk Potential of Games (SHARP-G) to assess addiction risk, supporting clinical diagnosis and forensic analysis of gaming-related incidents [34].

Neurophysiological evidence of gaming addiction

There are multiple studies conducted on the impact of gaming addiction on our neuropsychology. There is neurophysiological evidence describing an altered pattern of activation in behavioral addiction that consequently decreases learning processes [2,35,36]. Gaming addiction impairs reinforcement learning as evidenced by a reduced brain response to unexpectedly better rewards [35], mirroring cognitive deficits in internet addiction [3]. Furthermore, reversal learning is decreased and slower in gaming addicts [37]. Associative learning skills were clearly established to be reduced in individuals who had video gaming disorder [38].

Those individuals suffering from gaming disorder who frequently played first-person shooter games (such as Call of Duty and Overwatch) or those who massively played multiplayer online RPGs showed decreased working memory [35]. These results were associated with reduced functional integration within the left frontoparietal region and lower visuospatial working memory [35].

Gaming addicts exhibit heightened activation in brain regions tied to cognitive, emotional, and motor functions, including those responsible for decision-making, reward processing, attention, memory, and emotional regulation [39].

Risk factors for gaming addiction

Studies have shown that male gender, young age, social satisfaction, treatment need, and hours chatting over the internet/ social networks increase the probability of problematic or addictive games [2,40]. Certain personality traits like neuroticism, aggression and hostility, and sensation seeking are commonly associated with gaming and internet addiction [35].

Physiological and psychological symptoms of abnormal gaming behavior are both positively linked to the three substance abuse personality scales of the Minnesota Multiphasic Personality Inventory-2 (MMPI-2) [1]. The physical game-related symptoms also appear to be related to the psychological issues these youngsters experience from their problematic gaming behavior, rather than the hours played [1].

Psychosocial consequences of gaming addiction

Gaming addiction is shown to have the following psychological consequences, including sacrificing real-life relationships in favor of more gaming, decreased sleep, work, and education [35]. The syndrome of social withdrawal is best described by the Hikikomori syndrome, where an individual isolates themselves for more than six months, leading to significant functional impairment and/or distress [41]. Along with reduced attention, increased aggression and hostility, dysfunctional coping mechanisms, and memory issues, it is also associated with further psychosomatic sequelae, including sleep disorders and seizures [35].

Studies have also shown that anxiety and depression are common among those dependent on video games [35]. Lower self-esteem, lack of self-efficacy, reduced social support, and life satisfaction are also reported. Internet gaming addiction is linked to increased suicidal ideation and behavior [42], as evidenced by documented cases. Gaming addiction is also linked to decreased physical activity, neglected hygiene, and various medical issues resulting from continuous back strain, eye strain, and carpal tunnel syndrome [43].

A meta-analysis confirmed a moderate positive association between gaming disorder and aggression in teenagers and young adults, with the relationship being stronger in Asia, in the younger age group, and in more recent studies [44]. A longitudinal study of Korean adolescents revealed that gaming disorder and aggression mutually reinforced each other over the years, forming a vicious cycle in males [45]. In contrast, intrusive parenting and gaming disorder showed bidirectional reinforcement in both sexes [45]. Relationship of aggression and gaming disorder is multifactorial: male gender, age of the gamer, over-controlling parenting, level of education, family income, socioeconomic class, stress coping ability, peer victimization, type of game played (violent games), and family chaos [44,45,46]. Thus, the pathway from violent gaming to aggression is highly individual-specific and moderated by gender, pre-existing gaming problems, self-regulation, parenting style, and broader psychosocial context.

## Conclusions

Gaming addiction is a major issue that affects individuals’ lives, leading to behavioral abnormalities, as well as other problems. Examples of behavioral issues include suicide and violent actions, while other effects may involve decreased learning abilities, memory impairment, and sleep disturbances. Ultimately, gaming addiction poses a serious threat to both mental and physical well-being.
